# A Fenton-like method using ZnO doped MIL-88A for degradation of methylene blue dyes

**DOI:** 10.1039/d0ra08076d

**Published:** 2020-11-03

**Authors:** Gangli Ren, Ke Zhao, Lang Zhao

**Affiliations:** State Key Laboratory of Rare Earth Resource Utilization, Changchun Institute of Applied Chemistry, Chinese Academy of Sciences Changchun 130022 P. R. China zhaolang@ciac.ac.cn +86-431-85262878; Key Laboratory of Songliao Aquatic Environment, Ministry of Education, Jilin Jianzhu University Changchun 130118 P. R. China

## Abstract

MIL-88A with different sizes was prepared by hydrothermal method by changing the content of ZnO. The samples were characterized by SEM, TEM, XRD, XPS and FT-IR. The synthesized material was used for the removal of methylene blue dye in a Fenton-like reaction, and the optimal reaction conditions were studied through single factor experiments. The experimental results show that when the molar ratio of the amount of ZnO introduced to FeCl_3_·6H_2_O is 1 : 1, the obtained materials have better catalytic performance than others. Under the optimal conditions, MIL-88A(Fe_1_Zn_1_) has the best catalytic performance for 300 mg L^−1^ methylene blue. The removal rate can reach 96.15% within 40 minutes.

## Abstract 2

MIL-88A with different sizes were prepared by changing the content of ZnO. The prepared nanocomposites were used as catalysts in Fenton-like reaction of removing MB. The effects of the main reaction parameters on the reaction activity were studied.

## Graphical abstract

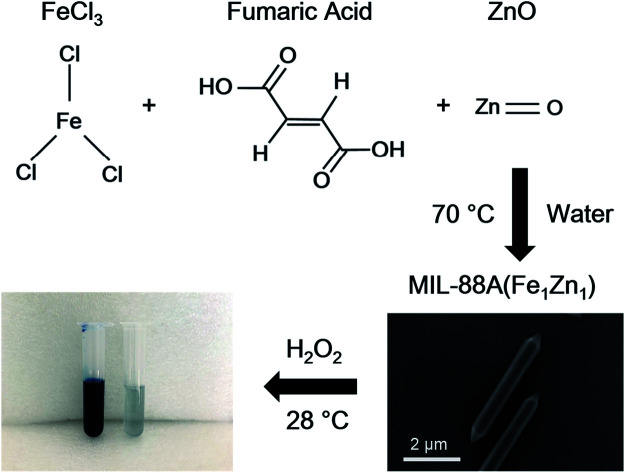

## Introduction

With the large-scale development of textile, printing and paper-making industries, the amount of waste water discharged in the production processes is increasing day by day. It is estimated that about 5–10% of commercial dyes will flow into the environment along with the discharge of industrial wastewater.^1^ The main characteristics of dye wastewater are darker color and low light transmittance, which will block sunlight penetration, thereby reducing the amount of dissolved oxygen and affecting the survival of aquatic animals and plants.^2,3^ Dye wastewater often contains highly toxic organic compounds, which have teratogenic, carcinogenic and lethal effects, and are not easily biodegraded.^4^ These wastewater, if not properly treated, may pose a significant threat to the ecosystem and human health. Therefore, it is urgently required to explore an effective method to treat dye wastewater to make the environment sustainable.

At present, the main methods of treating dye wastewater are adsorption,^5–7^ photocatalysis, membrane separation, biochemical technology, advanced oxidation processes (AOPs)^8–13^ and so on. Among these methods, AOPs is considered to be a promising technology that has attracted great attention. Due to its high efficiency and reproducibility, AOPs has been widely used in the removal of various harmful organic pollutants in wastewater. Fenton process as a typical advanced oxidation process has attracted great attention for removing refractory pollutants since it was discovered in 1894. Traditional homogeneous Fenton reaction is an efficient treatment method for degrading organic wastewater. In Fenton reaction system, Fe^2+^ reacts with H_2_O_2_ to generate Fe^3+^ and strongly oxidizing hydroxyl radicals (Fe^2+^ + H_2_O_2_ ∼ Fe^3+^ + ·OH + OH^−^), and then Fe^3+^ is reduced to Fe^2+^ (Fe^3+^ + H_2_O_2_ ∼ Fe^2+^ + ·O_2_H + H^+^) by reaction with H_2_O_2_. These two reactions are carried out cyclically, so hydroxyl radicals will be produced continuously.^14^ Unfortunately, the catalyst is easily deactivated due to iron leaching or low activity. For the traditional homogeneous Fenton reaction, there are still some inherent shortcomings: iron-containing sludge formation and unrecoverable catalyst.^15^ In order to overcome the disadvantages of homogeneous Fenton reaction, solid heterogeneous Fenton-like catalyst is used to activate hydrogen peroxide.^16^ At present, the most common heterogeneous Fenton-like catalyst is iron oxide, but the specific surface area of iron oxide is low, and the exposed active sites are limited, so its catalytic activity is low.^17,18^

Over the past two decades, metal organic frameworks (MOFs), a novel class of porous materials, have been widely studied in different research fields such as drug delivery, adsorption, gas storage, catalysis, luminescence and separation *etc.* MOFs are typically built from metal ions and organic linkers.^19–22^ Due to their fascinating properties such as large specific surface area, high porosity, flexible structure, tunable pore size, and thermal stability. MOFs, promising catalysts for dye wastewater treatment, have attracted considerable attention for their potential applications in catalysis.^23–25^ Li *et al.*^26^ reported a mesoporous uranium-based metal–organic framework and this material ultimately giving rise to the lowest-density MOF reported to date. Rimoldi *et al.*^27^ discussed some representative examples of replacing ZrMOFs with active materials. These catalysts often show higher performance or unusual activity compared with similar catalysts. Some studies have shown that iron-containing MOFs can be used as heterogeneous Fenton catalysts because of wide distribution of individual iron sites. However, the catalytic efficiency of pure MOFs still needs to be improved since these materials contain only Fe(iii) species with weak Fenton activity. Inorganic semiconductors such as ZnO are active catalysts because of their low-cost and non-toxicity.^28^ Therefore, constructing a heterojunction between MIL-88A(Fe) and ZnO may have great potential for the improving Fenton catalytic properties.

In this work, MIL-88A(Fe) was used as a host material to load different amounts of ZnO to synthesize a series of novel catalysts MIL-88A(Fe_1_Zn_*x*_) with high driven-Fenton catalytic activity. The obtained materials were characterized by SEM, TEM, XRD, XPS and FT-IR. Methylene blue (MB) was used as a target contaminant to investigate the Fenton catalytic activity of MIL-88A(Fe). The influence of reaction parameters, such as initial MB concentration, H_2_O_2_ concentration, pH and temperature, were investigated by Single factor experiment method. The stability and reusability of the materials were also tested. Additionally, the possible reaction mechanisms involved in the Fenton system were discussed.

## Experimental

### Chemicals and materials

All chemicals in this study were analytical grade and used without further purification. FeCl_3_·6H_2_O was purchased from Macklin Biochemical Co., Ltd. (Shanghai, China). Fumaric acid was obtained from Tianjin Guangfu Fine Chemical Research Institute. ZnO was purchased from Beijing Yili Fine Chemical Co., Ltd. Methylene blue (MB) was purchased from Sinopharm Chemical Reagent Co., Ltd. H_2_O_2_ (30%) was purchased from Beijing Chemical Works. T-butyl alcohol (TBA) was bought from the Xilong Scientific Co., Ltd. (Shantou, China). Ultrapure water was used throughout the experiments.

### Synthesis of catalyst

The synthesis of MIL-88A(Fe) was performed *via* a hydrothermal method.^29–31^ Typically, FeCl_3_·6H_2_O (5 mmol) and fumaric acid (5 mmol) were dissolved in 40 mL deionized water respectively. Then, the solution of FeCl_3_·6H_2_O was added to the fumaric acid solution, it was stirring for 4 h at room temperature. Finally, the reaction mixture was transferred into a Teflon-lined stainless steel autoclave and heated to 70 °C for 12 h. After cooling to room temperature, the resulting products were washed with deionized water repeatedly and dried at 80 °C for 6 h in a vacuum oven after centrifugation. The obtained powder was named MIL-88A(Fe).

The ZnO doped MIL-88A(Fe) is obtained by adding ZnO powders with different proportions in the preparation process, and other steps are the same as above. According to the different amounts of zinc oxide: 2.5 mmol, 5 mmol and 7.5 mmol, the samples were labeled as MIL-88A(Fe_1_Zn_0.5_), MIL-88A(Fe_1_Zn_1_) and MIL-88A(Fe_1_Zn_1.5_) respectively.

### Characterization

The morphology and microstructure of the samples were examined by using scanning electron microscopy (SEM, Qunta250) with an accelerating voltage of 10 kV. Transmission electron microscopy (TEM) images were recorded on a FEI Tecnai G2 F20 instrument (FEI company, Netherlands). The X-ray powder diffraction (XRD) patterns were studied using a D8-Focus diffractometer (Bruker) in the 2*θ* range from 5° to 80°, using Cu target as the radiation source (40 kV, 40 mA). The elemental composition and phase structures were recorded by X-ray photoelectron spectroscopy (XPS) performed using an AXIS Ultra instrument (Kratos Analysis Company). The Fourier transform infrared (FT-IR) spectra were collected within the 400–4000 cm^−1^ spectral range on a Thermo Fisher Nicolet-6700 spectrometer. The ultraviolet-visible spectroscopy measurements were carried out on a UV-3600 UV-vis spectrophotometer (Shimadzu).

### Fenton-like catalytic experiments

Methylene Blue (MB) was selected as a target pollutant to investigate MIL-88A (Fe) catalytic activity. The degradation experiments were carried out in a batch mode using beaker (50 mL), and the pH of methylene blue solution was adjusted with HCl (0.1 mol L^−1^) or NaOH (0.1 mol L^−1^). In a typical experiment, H_2_O_2_ was first added to MB solution, and then the degradation reaction was initiated by adding catalyst under ultrasonic condition. At predetermined time intervals, samples were withdrawn and filtered through 0.22 μm membrane filters to remove suspended MOFs. Meanwhile, an aliquot of *tert*-butyl alcohol (TBA) was immediately added to quench the reaction. The remaining concentration of MB was analyzed using an ultraviolet-visible spectrophotometer at 664 nm. The decolorization efficiency of MB was calculated as follows equation:
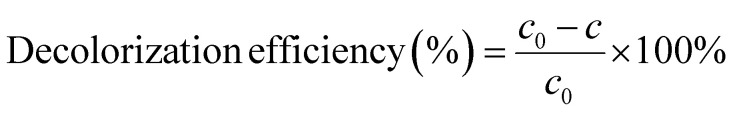
where *c*_0_ is the initial concentrations of MB solution, *c* is the dye concentration at certain time during the Fenton reaction process.^32^ Each MB degradation experiment was duplicated and repeated at least twice. The reported data are arithmetic means of measured results.

In the test of catalyst repeatability, the catalyst after reaction was collected and washed several times with deionized water, and then reused in a new reaction after being dried in a vacuum drying box at 80 °C. This process was repeated several times to test the reusability of catalyst. The performance of H_2_O_2_ oxidation without MIL-88A(Fe) and the amount of MB adsorbed by MIL-88A(Fe) were tested as control experiments.

## Results and discussion

### Synthesis and characterization

**Fig. 1:**
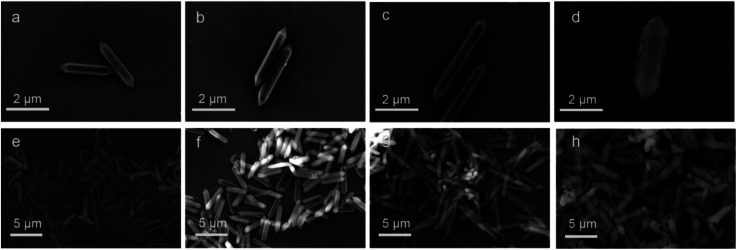
SEM images of (a and e) MIL-88A(Fe), (b and f) MIL-88A(Fe_1_Zn_0.5_), (c and g) MIL-88A(Fe_1_Zn_1_) and (d and h) MIL-88A(Fe_1_Zn_1.5_).

**Fig. 2:**
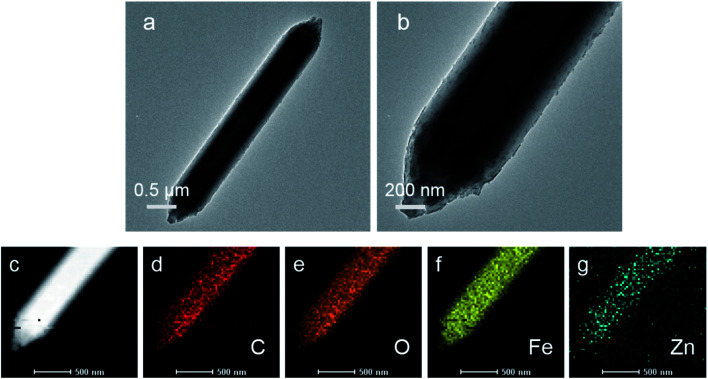
TEM images of (a and b) MIL-88A(Fe_1_Zn_1_) and (c–g) EDS elemental mapping of MIL-88A(Fe_1_Zn_1_).

The microstructure and elemental composition of the samples were studied by scanning electron microscope and transmission electron microscope. Images of MIL-88A(Fe), MIL-88A(Fe_1_Zn_0.5_), MIL-88A(Fe_1_Zn_1_) and MIL-88A(Fe_1_Zn_1.5_) are shown in the Fig. 1, 2(a and b). The first three catalysts are hexagonal rods with complete structure and uniform size. The length of the catalysts changed with the increase of ZnO content, when the molar ratio of Fe and Zn is 1 : 1, the crystal length reaches the maximum. The average size of MIL-88A(Fe_1_Zn_1_) is about 4.5 μm. Due to the introduction of more ZnO, only a few complete crystals of MIL-88A(Fe_1_Zn_1.5_) were formed. Most of them are in a fragmented state with different sizes and adhesive structures. It can be seen that doping ZnO does not obviously change the original crystal morphology of the catalyst, but has certain influence on its size. The nucleation rate of MOF determines its morphology or particle size. Generally, rapid nucleation shortens the crystal growth stage, resulting in small-sized particles. On the contrary, slow nucleation will produce large-sized particles.^33^ It can be speculated that the introduction of ZnO may affect the nucleation rate of materials, resulting in crystals with different sizes. The element energy dispersive X-ray spectrum of MIL-88A(Fe_1_Zn_1_) is shown in the Fig. 2(c–g). It can be seen that the C, O, Fe, and Zn elements are distributed uniformly in the material, which also directly proves the successful doping of ZnO.

**Fig. 3:**
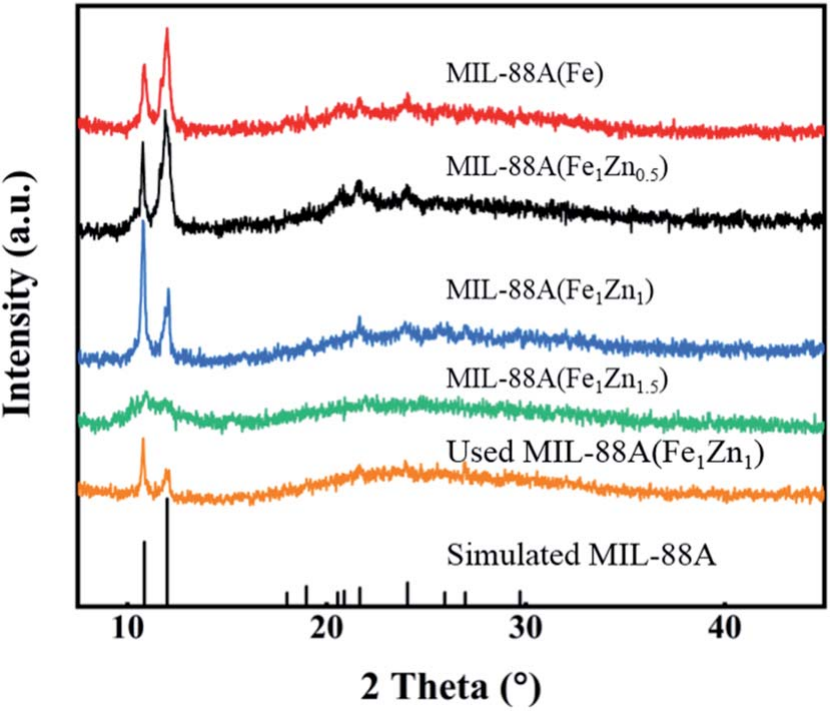
XRD patterns of MIL-88A(Fe), MIL-88A(Fe_1_Zn_*x*_) and used MIL-88A(Fe_1_Zn_1_).

Fig. 3 shows the XRD spectra of four catalysts, and the existence of sharp diffraction peaks confirms the crystalline properties of the products. It can be seen from the figure that with the different amount of doped ZnO, the corresponding XRD image also changes. Except MIL-88A(Fe_1_Zn_1.5_), the diffraction peak positions of MIL-88A(Fe) and MIL-88A(Fe_1_Zn_*x*_) are basically the same, and both have strong diffraction peaks at 2 theta = 10.79° and 11.98°, which correspond to the (100) and (101) planes of MIL-88A respectively.^34^ However, with the increase of ZnO doping amount, the relative intensity of these two diffraction peaks changes. The peak intensity ratios of MIL-88A(Fe), MIL-88A(Fe_1_Zn_0.5_) and MIL-88A(Fe_1_Zn_1_) are 37 : 63, 40 : 60 and 70 : 30, respectively. It can be inferred that the introduction of ZnO has a certain function in controlling the crystal orientation of MIL-88A. It shows that doping ZnO may change the dominant crystal plane exposed by the material. Liao *et al.*^35^ confirmed that the (100) surface of MIL-88A-Fe showed lower energy barrier for H_2_O_2_ dissociated into ·OH and the exposed (100) ratio determined its catalytic performance.

**Fig. 4:**
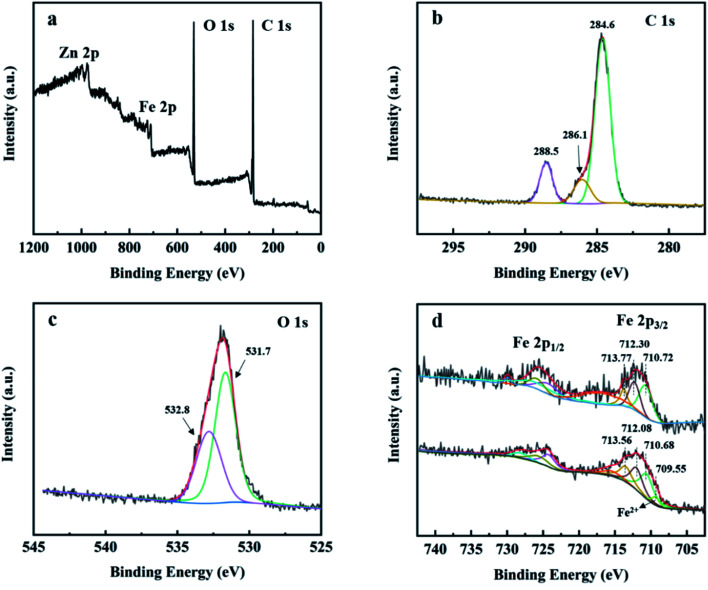
XPS survey spectrum of (a) high resolution XPS spectra, (b) C 1s, (c) O 1s and (d) fresh MIL-88A(Fe_1_Zn_1_) and used MIL-88A(Fe_1_Zn_1_).

X-ray photoelectron spectroscopy (XPS) was used to study the surface elemental composition of MIL-88A and the chemical state of iron ions on the catalyst surface before and after reaction. In Fig. 4(a), the XPS survey spectrum verified that C, O, Fe and Zn elements exist in MIL-88A(Fe_1_Zn_1_). As shown in Fig. 4(b), the C 1s spectrum of MIL-88A(Fe_1_Zn_1_) was deconvoluted to three peaks located at 288.5, 286.1 and 284.6 eV. Specifically, the peaks can be attributed to carboxylic bands and benzoic rings respectively.^35,36^ As shown in Fig. 4(c), there are two peaks at 532.8 and 531.7 eV for the O 1s spectrum of MIL-88A(Fe_1_Zn_1_), which could ascribed to the oxygen components on the organic linkers and the Fe–O bonds of Fe-MOF, respectively.^35,37^ It is generally believed that the electron exchange between Fe(ii)/Fe(iii) and H_2_O_2_ can induce the formation of hydroxyl radicals in homogeneous and heterogeneous Fenton-like reactions.^38^Fig. 4(d) shows the high-resolution scanning XPS spectra of Fe 2p in fresh and reacted MIL-88A(Fe_1_Zn_1_), which are unrolled into multiple peaks by Gaussian curves. Fresh MIL-88A(Fe_1_Zn_1_) shows spin orbits of 725.7 eV (Fe 2p_1/2_) and 712.1 eV (Fe 2p_3/2_) with peak distances of about 13.6 eV.^37^ The Fe 2p_3/2_ peak can be fitted to three contribution values of 710.72 eV, 712.30 eV and 713.77 eV respectively, which indicates that only Fe(iii) exists on the fresh MIL-88A(Fe_1_Zn_1_). The weak oscillation satellite peaks of about 717.1 eV observed in the Fe 2p spectra further confirm that iron is mainly in the Fe(iii) state.^36,39,40^ After Fenton-like reaction, the binding energy of Fe 2p decreased slightly to 724.5 eV and 712.0 eV, respectively. After deconvolution of the Fe 2p_3/2_ peak of MIL-88A(Fe_1_Zn_1_), the Fe(ii) peak of MIL-88A(Fe_1_Zn_1_) was found at 709.55 eV, and the satellite peak of 717.1 eV disappeared, indicating that part of Fe(iii) was reduced to Fe(ii) during Fenton-like reaction.^41^

**Fig. 5:**
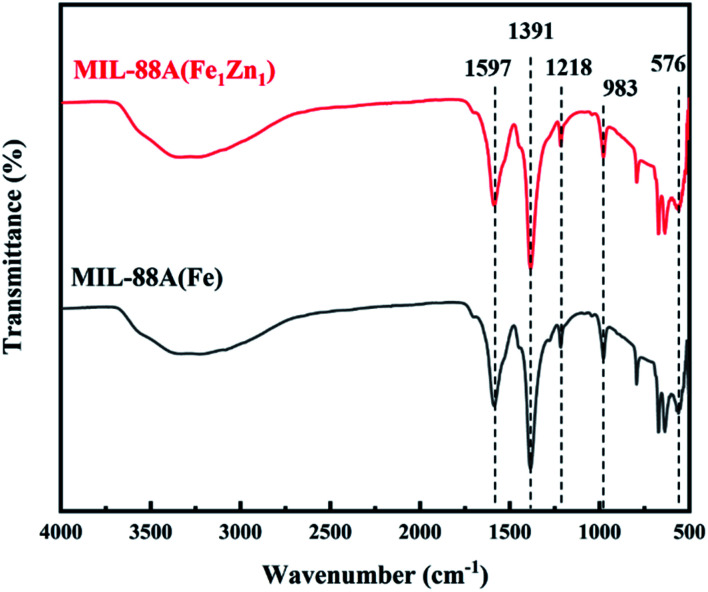
FT-IR of MIL-88A(Fe) and MIL-88A(Fe_1_Zn_1_).

The FT-IR spectrum of MIL-88A(Fe) and MIL-88A(Fe_1_Zn_1_) composite samples are shown in Fig. 5. Owing to the water adsorption of samples, there is a wide bulge at a higher wave number between 3000–3500 cm^−1^. The peaks detected at 1597 cm^−1^ and 1391 cm^−1^ are attributed to the symmetric and asymmetric vibration modes of the fumarate carboxyl group as the MOF bridging ligand C=O group vibration.^42^ The characteristic peaks at 1218, 983 and 576 cm^−1^ are respectively attributed to the stretching vibration of C–C, the bending vibration of C–H and the stretching vibration of Fe–O.^43^ From these results, one can find out that the FT-IR spectra of MIL-88A(Fe) and MIL-88A(Fe_1_Zn_1_) are identical.

### Factors affecting the catalytic activity of methylene blue in Fenton like oxidation

In order to further understand the influence of the prepared catalyst on MB decolorization in heterogeneous Fenton-like reaction, single factor experiment method was used to explore the conditions affecting the reaction process, and it was found that MIL-88A(Fe_1_Zn_1_) had the best catalytic effect under the optimum conditions, and the removal rate of MB with a concentration of 300 mg L^−1^ could reach over 96% within 40 minutes.

**Fig. 6:**
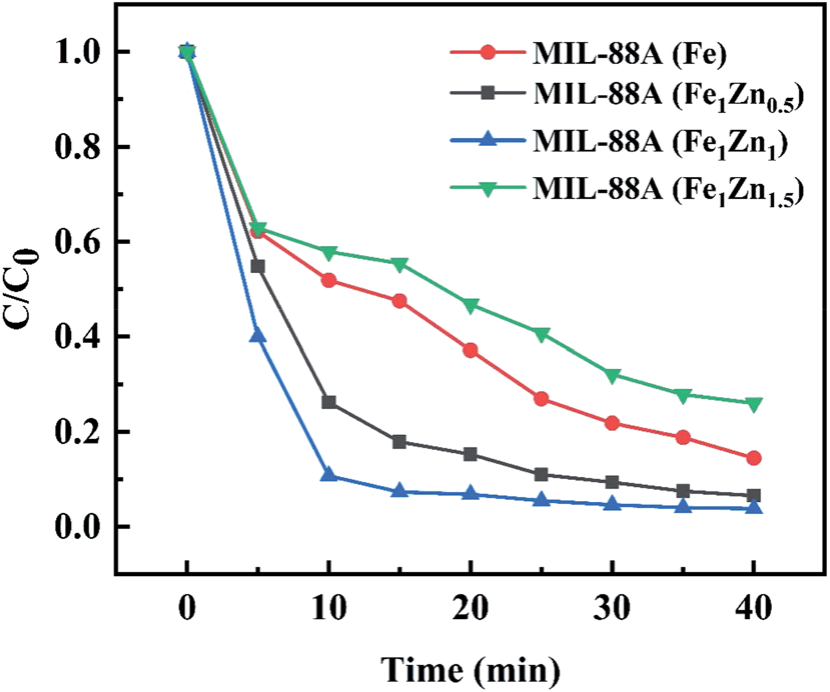
Degradation of MB under different reaction conditions (reaction conditions: H_2_O_2_, 2.5 mL L^−1^; pH, 3; temperature, 28 °C; initial MB concentration 300 mg L^−1^; catalyst, 1 g L^−1^).

Under the conditions of MB concentration of 300 mg L^−1^, catalyst dosage of 1 g L^−1^, H_2_O_2_ concentration of 2.5 mL L^−1^, initial pH of 3 and temperature of 28 °C, the influence of ZnO doping amount on the catalytic activity of MIL-88A was investigated. The results are shown in the Fig. 6, the MB removal rate of MIL-88A(Fe), MIL-88A(Fe_1_Zn_0.5_), MIL-88A(Fe_1_Zn_1_) and MIL-88A(Fe_1_Zn_1.5_) are 85.51%, 93.42%, 96.15% and 73.96% respectively. It can be seen that increasing the doping amount of ZnO within a certain range can improve the effect of MIL-88A catalyzed Fenton-like reaction to treat pollutants. However, the introduction of excessive ZnO has a great influence on the catalytic effect, and MIL-88A(Fe_1_Zn_1_) has the highest catalytic activity, which may be due to the fact that ZnO at this ratio can more promote the conversion between trivalent iron and divalent iron. In the following experiments, MIL-88A(Fe_1_Zn_1_) was used as a catalyst for research.

**Fig. 7:**
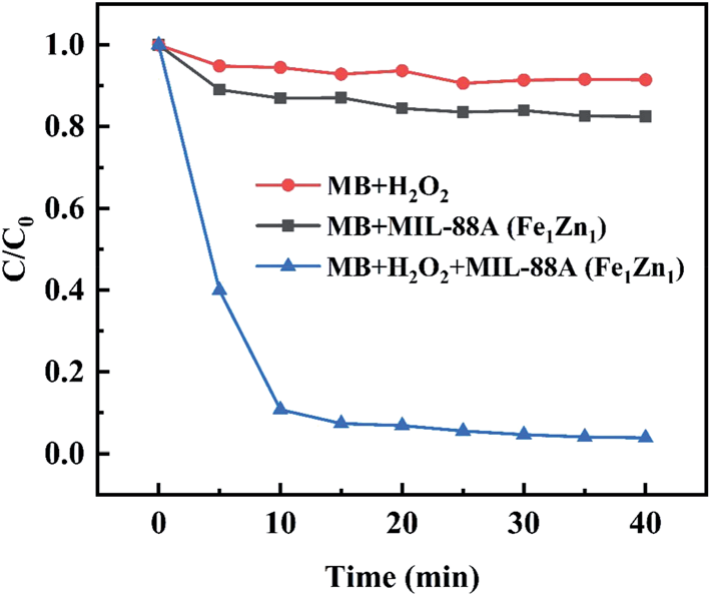
Removal of MB in different processes (control experiments). Other conditions are the same as in Fig. 6.

Results as shown in the Fig. 7, in the presence of H_2_O_2_ alone, the concentration of MB hardly changed after 40 minutes of reaction, which indicated that the ability of oxidative decomposition of MB by H_2_O_2_ alone was weak. When only the catalyst is added to participate in the reaction, the concentration of MB decreases slightly due to the adsorption effect of the catalyst, but because the specific surface area of the catalyst MIL-88A(Fe_1_Zn_1_) is not developed, the removal rate of MB in the reaction time is only about 18%, which proves that MB can't be removed only by adsorption mechanism. From the above experiments, it can be concluded that when H_2_O_2_ and catalyst are added to MB respectively, MB can't be effectively decolorized within the specified reaction time, so it is possible to rule out their separate roles in heterogeneous Fenton-like reaction.

**Fig. 8:**
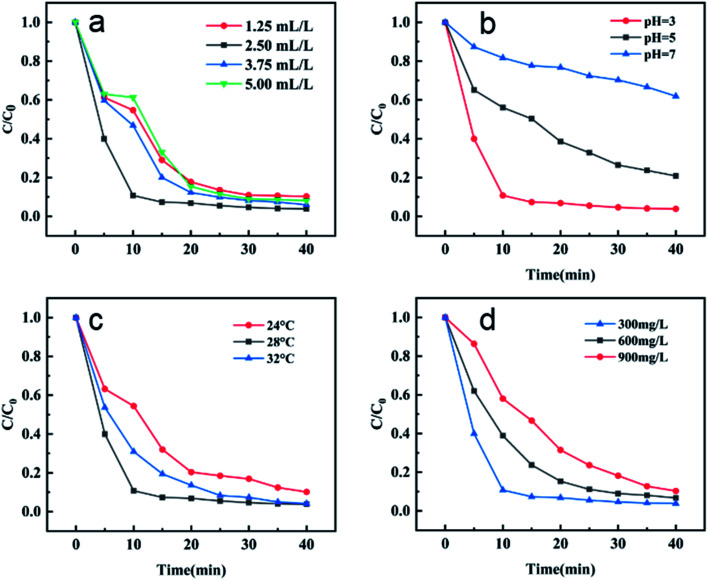
The effect of various parameters on the MB degradation in MIL-88A(Fe_1_Zn_1_) catalyzed Fenton-like system: (a) H_2_O_2_ concentration, (b) initial pH value, (c) reaction temperature, and (d) initial MB concentration. Except for the investigated parameter, other conditions are the same as in Fig. 6.

For Fenton-like reaction, the amount of H_2_O_2_ is one of the main factors affecting the reaction process. Excessive H_2_O_2_ will bring hydroxyl radicals, which will reduce the speed of MB degradation by catalysts. Adding a large amount of H_2_O_2_ is also a waste of raw materials; while the amount of H_2_O_2_ is insufficient, the hydroxyl radical produced by H_2_O_2_ is insufficient, which will greatly reduce the degradation effect. Therefore, it is necessary to investigate the most suitable amount of H_2_O_2_ in Fenton-like reaction. The results are shown in Fig. 8(a), when the initial concentration of MB is 300 mg L^−1^, 20 mL, the temperature is 28 °C, the dosage of catalyst MIL-88A(Fe_1_Zn_1_) is 1 g L^−1^ and the initial pH is 3, the most suitable amount of H_2_O_2_ is 2.5 mL L^−1^. Within the reaction time, the removal rates of MB with H_2_O_2_ of 1.25 mL L^−1^, 2.5 mL L^−1^, 3.75 mL L^−1^ and 5 mL L^−1^ were 89.7%, 96.15%, 94.0% and 91.9%, respectively. Although there was no significant difference in the final removal efficiency between the four groups, it took only 15 minutes for the removal rate of “2.5 mL L^−1^” group to basically stabilize, while it takes 25 minutes or more for the other three groups. In addition, in order to follow the principle of saving raw materials, under the condition of ensuring MB removal rate, a smaller amount of H_2_O_2_ was selected for the experiment.

PH is also an important factor affecting Fenton-like reaction. As shown in the Fig. 8(b), the MB removal efficiency of the catalyst at pH 3, 5 and 7 was investigated respectively. It can be seen that the catalytic efficiency at pH 3 is about 96.15%, which is obviously better than others. Under the same experimental conditions, the catalytic efficiency decreased significantly when the pH is further increased to 5, reaching only 79.17%. By changing the pH value and conducting experiments under neutral conditions, the removal rate is further reduced to 38.08%.

As we all know, temperature change has a vital influence on the catalytic reaction. Generally speaking, higher reaction temperature is beneficial to the forward progress of the catalytic process, but higher temperature will bring more energy consumption, so it is an essential step to find a more suitable reaction temperature. In this experiment, 24 °C, 28 °C and 32 °C were selected as the reaction temperature. It can be seen from Fig. 8(c), with the increase of reaction temperature, the removal efficiency of MB by the catalyst is also greatly improved, with the removal rates being 89.86%, 96.15% and 95.83%, respectively. When the temperature rises from 24 °C to 28 °C, the removal rate increases by nearly 7%. However, when the temperature continues to rise, the removal rate did not increase further. In addition, considering the advantages of energy saving, convenience and trouble saving, the experiment was carried out at room temperature as far as possible. To sum up the above, 28 °C was chosen as the optimum temperature for the experiment.

Most reports on removing dyes by Fenton or Fenton-like reaction generally focus on the study of low dye concentration: 5 mg L^−1^–20 mg L^−1^, and the study of medium and high dye concentration is rare, even when the dye concentration is as high as 500 mg L^−1^, the degradation process takes a lot of time. In this study, three different concentrations of MB were selected as treatment objects to explore the influence of initial MB concentration on the reaction. It can be seen from the Fig. 8(d), with the dye concentration increasing exponentially from 300 mg L^−1^ to 600 mg L^−1^ and then to 900 mg L^−1^, the MB removal efficiency of the catalyst does not decrease proportionally, but shows a relatively mild downward trend, with removal rates of 96.15%, 93.28% and 89.69% respectively. It can be seen that MIL-88A(Fe_1_Zn_1_) can still show a good removal effect in a short reaction time even when the initial concentration of MB is very high.

The comparison of the catalytic activity between MIL-88A(Fe_1_Zn_1_) and various previous reported catalysts for the degradation of pollutants are listed in Table 1. The results show that the performance of MIL-88A(Fe_1_Zn_1_) catalyst is superior to those reported in the literature, especially when the higher concentrations of dye and reaction time in our work are taken into consideration. In general, with smaller dye concentration, the degradation rate increases due to more available hydroxyl radicals and their interaction with organic molecules. However, we used higher initial concentrations of the selected dye to assess the activities of MIL-88A(Fe_1_Zn_1_) catalyst. Obviously, MIL-88A(Fe_1_Zn_1_) prepared in this study has high catalytic activity. It can be used to treat high concentration in a shorter time, which indicates that MIL-88A(Fe_1_Zn_1_) is a potential catalyst for the degradation of MB.

**Table 1:** Comparison of the catalytic activity of the MIL-88A(Fe_1_Zn_1_) and related catalysts for MB

Catalyst	Target pollutants	Amount (mg L^−1^)	Catalyst used (g L^−1^)	Time (min)	Decolorization efficiency (%)	Ref.
Degussa P-25	RhB	47.9	1	70	45	2
ZnO/MIL-101(Fe)	RhB	10	0.5	300	97.1	3
CUS-MIL-100(Fe)	SMT	20	0.5	180	98.4	19
ZnO/ZIF-9	TC	10	0.2	60	87.7	20
MIL-88A	RhB	10	0.5	120	80	35
RGO-MOF-ZnO	MB	10	1	120	82	44
MIL-88A(Fe_1_Zn_1_)	MB	300	1	40	96.15	This work
MIL-88A(Fe_1_Zn_1_)	MB	600	1	40	93.28	This work
MIL-88A(Fe_1_Zn_1_)	MB	900	1	40	89.69	This work

**Fig. 9:**
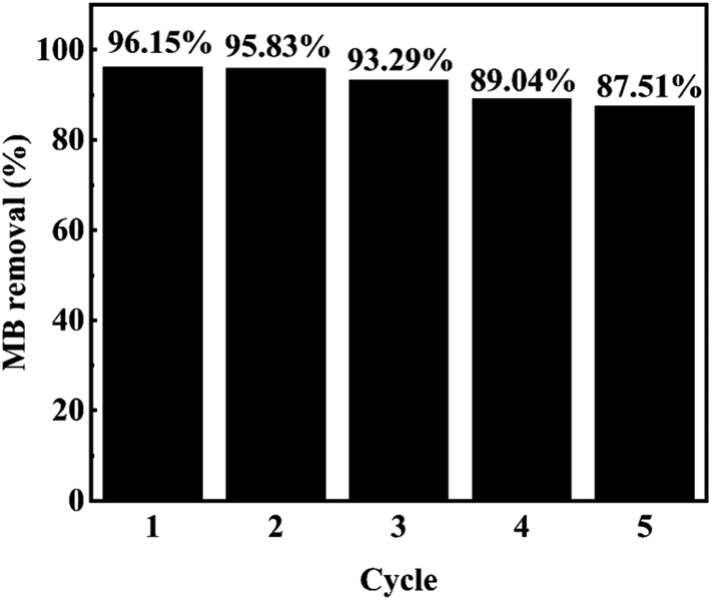
Removal efficiency of MB during the degradation of MB in five consecutive runs. (Other conditions are the same as in Fig. 6).

In addition to the catalytic activity of the catalyst, the stability and reusability of the catalyst are also important indexes to investigate the performance of the catalyst. As shown in the Fig. 9, in five consecutive repeated experiments, the removal rates of methylene blue by H_2_O_2_ catalyzed by MIL-88A(Fe_1_Zn_1_) were still above 85%. The degradation rate of the MB with MIL-88A(Fe_1_Zn_1_) decreased a little every time, which may be caused by the mass loss of the catalyst in the process of recovering and iron leaching during the reaction process. The catalytic efficiency of MIL-88A(Fe_1_Zn_1_) remains basically unchanged, indicating that it has high stability and can be reused. In addition, the crystal structure of MIL-88A(Fe_1_Zn_1_) before and after reactions were further examined by XRD analysis, as shown in Fig. 3. The results of XRD revealed that the catalyst had no obvious transformation during the Fenton-like reaction process, which demonstrated that MIL-88A(Fe_1_Zn_1_) had great structural stability.

**Fig. 10:**
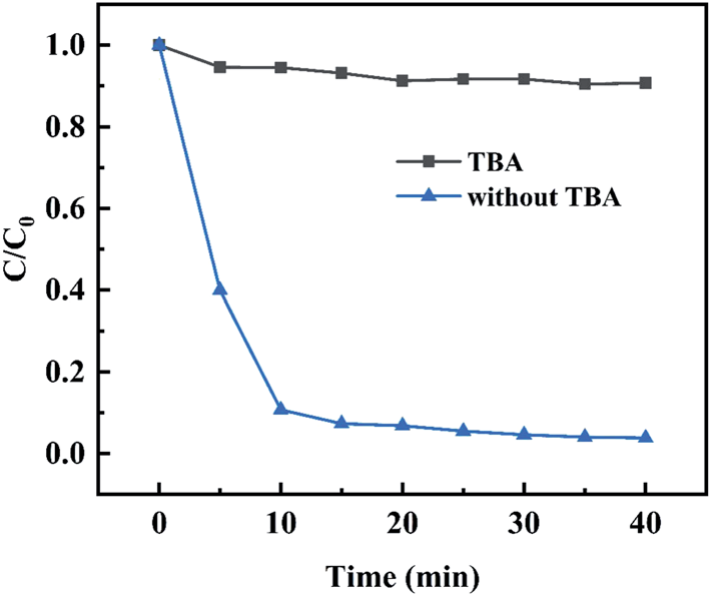
Inhibitory effect of TBA on MB degradation catalyzed by MIL-88A(Fe_1_Zn_1_).

With TBA as a free radical quencher, the influence of hydroxyl radicals generated in the system on MB degradation efficiency was investigated. As shown in the Fig. 10, when 20 μL TBA was added into the reaction system, the degradation efficiency of MB was almost completely inhibited, and its removal efficiency was only about 10% within the reaction time, which indicated that hydroxyl radicals were the main active substances for MB degradation.

The coordination unsaturated iron atom is the active center of MIL-88A(Fe_1_Zn_1_) catalyzed Fenton-like reaction, and its direct contact with H_2_O_2_ is the key step. First, H_2_O_2_ attaches to the iron site because H_2_O_2_ is a strong Lewis base with a stronger affinity for the iron site than water and unbridged anionic ligands, and then the electron/charge transfer between H_2_O_2_ and MIL-88A(Fe_1_Zn_1_), Fe(iii) can be reduced to Fe(ii) on MIL-88A(Fe_1_Zn_1_), then Fe(ii) formed on the surface can activate H_2_O_2_ reaction to form hydroxyl radical, organic pollutants are oxidized by surface-bonded hydroxyl radicals or by some hydroxyl radicals dispersed in mixed solutions. Some trivalent iron in MIL-88A(Fe_1_Zn_*x*_) can be converted to divalent iron by doping ZnO, and the activity of the catalyst can be improved by increasing the content of divalent iron.

## Conclusions

In this paper, a novel material MIL-88A(Fe_1_Zn_1_) was successfully synthesized and used as heterogeneous Fenton-like catalyst for the treatment of MB. Single factor experiment methods were employed to screen and optimized the variables which influenced the degradation of MB. Under the optimal conditions, MIL-88A(Fe_1_Zn_1_) exhibited high catalytic activity in the Fenton system and 96.15% of MB decolorization efficiency was achieved. It shows that doping ZnO may change the dominant crystal plane exposed by the material. The superior activity of MIL-88A(Fe_1_Zn_1_) was mainly attributed to the abundance of coordinatively unsaturated irons, the lower energy barrier resulting from the prominent (100) surface, and the enhanced Fe(iii)/Fe(ii) redox properties. The reactive species identification experiments revealed that hydroxyl radical play a key role in the reaction process. In addition, the recycling experiments also indicated that the as prepared catalyst had excellent stability, certifying that the composites are potential candidates for pollutant degradation.

## Conflicts of interest

There are no conflicts to declare.

## Supplementary Material
